# The genus *Eutreptiella* (Euglenophyceae/Euglenozoa) across its global distribution range

**DOI:** 10.1002/ece3.70241

**Published:** 2024-09-05

**Authors:** Sinuhé Hernández Márquez, María Eugenia Zamudio‐Resendiz, Montserrat Murrieta‐Alarcón, María Luisa Núñez Resendiz, Kurt M. Dreckmann, Abel Sentíes

**Affiliations:** ^1^ Doctorado en Ciencias Biológicas y de la Salud Universidad Autónoma Metropolitana Ciudad de Mexico Mexico; ^2^ Laboratorio de Fitoplancton Marino y Salobre, Departamento de Hidrobiología Universidad Autónoma Metropolitana Iztapalapa Mexico; ^3^ Laboratorio de Macroalgas Marinas y Salobres, Departamento de Hidrobiología Universidad Autónoma Metropolitana Iztapalapa Mexico

**Keywords:** biofuel production, biotechnological potential, *Eutreptiella*, genetic variability, marine ecosystems, photosynthesis

## Abstract

The genus *Eutreptiella* (Euglenophyceae/Euglenozoa) comprises unicellular organisms known for their photosynthetic capacity and significant role in marine ecosystems. This review highlights the taxonomic, ecological, and biotechnological characteristics of *Eutreptiella* species, emphasizing their morphological and genomic adaptations. *Eutreptiella* species exhibit high phenotypic plasticity, enabling adaptation to various environmental conditions, from nutrient‐rich waters to high‐salinity conditions. They play a crucial role in primary production and nutrient cycling in marine ecosystems. Genetic and transcriptomic studies have revealed their complex regulatory mechanisms and potential for biofuel and nutraceutical production. *Eutreptiella* blooms significantly impact local ecosystems, influencing nutrient availability and community dynamics. Additionally, interactions with associated bacteria enhance their growth and metabolic capabilities. The genus shows substantial genetic variability, suggesting potential misidentifications or a polyphyletic nature. Further comprehensive studies are needed to clarify their taxonomy and evolutionary relationships. Understanding and managing *Eutreptiella* populations is essential to leverage their biotechnological potential and ensure the health of marine ecosystems.

## Graphical abstract

The genus *Eutreptiella* across its global distribution range.

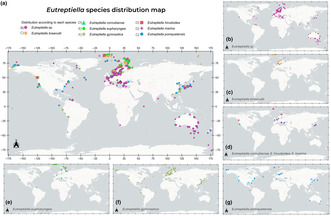

## 1 INTRODUCTION

**FIGURE 1:**
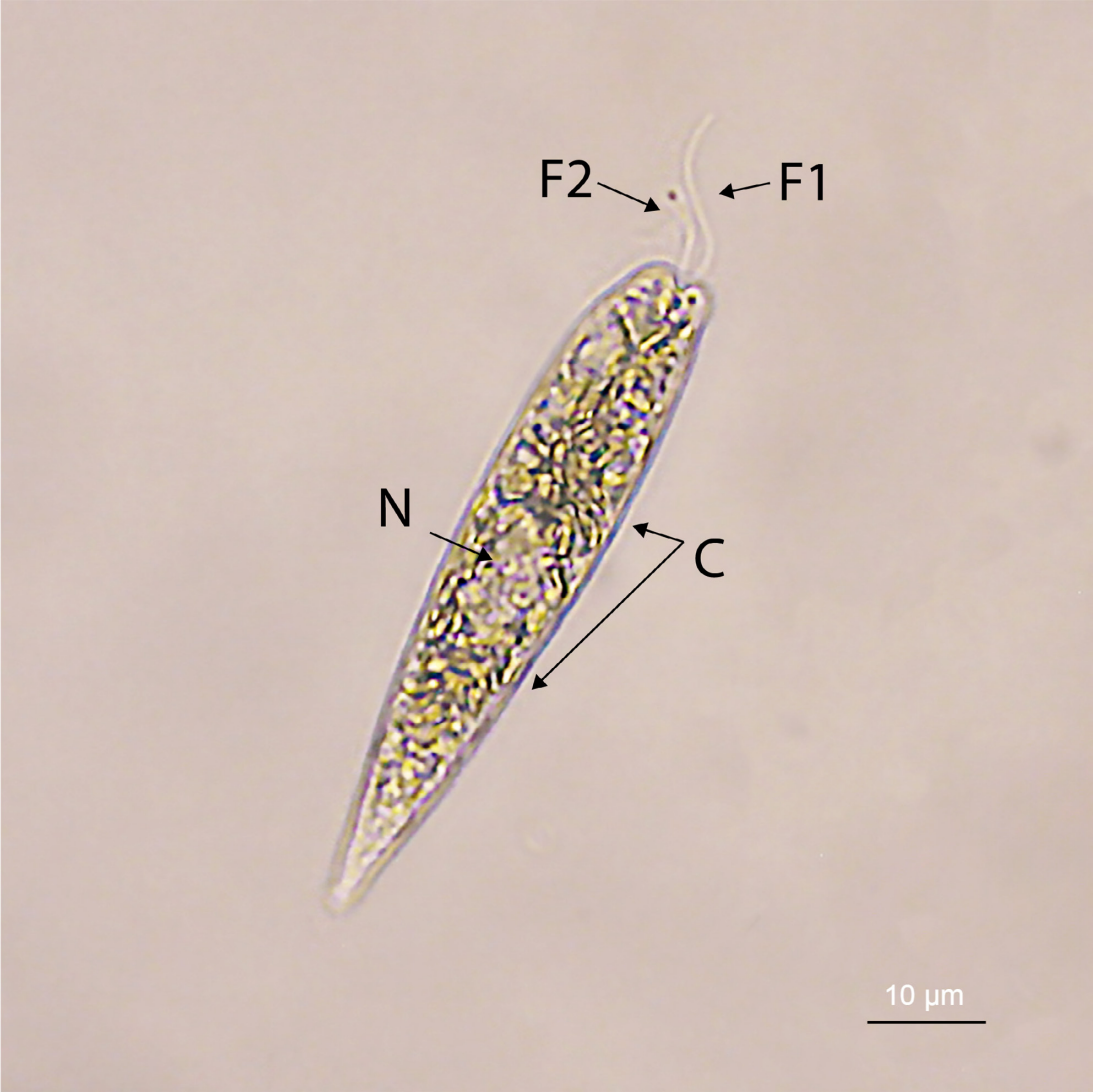
Overview of an organism of the species *Eutreptiella* sp. (F1 and F2) Two emergent unequal flagella, (C) chloroplast, (N) nucleus.

The genus *Eutreptiella* A.M. Da Cunha comprises a group of unicellular organisms belonging to the class Euglenophyceae within the phylum Euglenozoa (Figure 1). It includes nine taxonomically accepted species: *E. braarudii* Throndsen, *E. cornubiense* Butcher, *E. dofleinii* (Schiller) Pascher, *E. elegans* (Schiller) Pascher, *E. eupharyngea* Moestrup & R.E. Norris, *E. gymnastica* Throndsen, *E. hirudoidea* Butcher, *E. marina* A.M. Cunha, and *E. pomquetensis* (McLachlan, Seguel & Fritz) Marin & Melkonian (Guiry & Guiry, 2024). These species are known for their ability to perform photosynthesis and play an important role in marine ecosystems as primary producers. Since its circumscription in 1913 by Da Cunha, *Eutreptiella* has captured scientific interest due to its ecological importance and potential in biotechnological applications (Da Cunha, 1913; Hrdá et al., 2012). This review aims to highlight the taxonomic, ecological, and biotechnological characteristics of the *Eutreptiella* species, as well as their distribution and morphological and genomic adaptations.

## 2 MORPHOLOGICAL CHARACTERISTICS

Species of *Eutreptiella* are characterized by having two or more unequal flagella, a stigma, and discoidal, stellate, or reticulate chloroplast grouped according to the species. These organisms exhibit metabolic movements that allow them to adapt to various environmental conditions. This movement capability is facilitated by their flexible structure and their ability to modify their cell shape (Farutin et al., 2013). They possess photosynthetic capacity, making them essential components of phytoplankton, significantly contributing to biomass in marine ecosystems (Zamudio‐Resendiz et al., 2023).

Over the years, the genera *Eutreptiella* and *Eutreptia* Perty have been consistently studied together due to their morphological similarities. The primary variation between them lies in their flagella: The species of *Eutreptia*, in contrast to *Eutreptiella*, have two isokont flagella (Wang et al., 2021).

The distinctive morphology of *Eutreptiella* species facilitates their identification in taxonomic studies. The stigma, a light‐sensitive pigmented organelle, allows these algae to orient their movements toward light sources, optimizing photosynthesis. The chloroplasts contain photosynthetic pigments, primarily chlorophylls *a* and *b*, essential for capturing solar radiation and converting solar energy into chemical energy (Alfayate et al., 2008; Walne et al., 1986). Detailed studies of their morphology have revealed specific adaptations that enable their survival and proliferation in marine and brackish environments. These adaptations include the ability to perform rapid and precise movements as well as the formation of cysts under unfavorable conditions, allowing them to withstand periods of environmental stress (Kang et al., 2023; Olli, 1996).

## 3 PHYLOGENY AND TAXONOMY

Currently, the only way to molecularly identify and perform evolutionary comparisons of *Eutreptiella* species is through the 18S or SSU rRNA gene, due to the available information. Although other genes are known, they have been found unsuitable for species‐level differentiation studies. Therefore, in 2003, Marín et al. decided to modify the genus diagnosis to the following: “Organisms with two or four flagella of unequal length; SSU rRNA molecule encoded in the nucleus typically with the loop of helix 18 having a two‐nucleotide insertion (CA) after the second position, and typically in helix 38, the fourth‐last base pair is A‐U; distributed mainly in marine habitats.” Additionally, *Gymnastica* Schiller 1925 and *Tetreutreptia* McLachlan, Seguel & Fritz 1994 are considered synonyms, and the type species is *Eutreptiella marina* (Guiry & Guiry, 2024).

**FIGURE 2:**
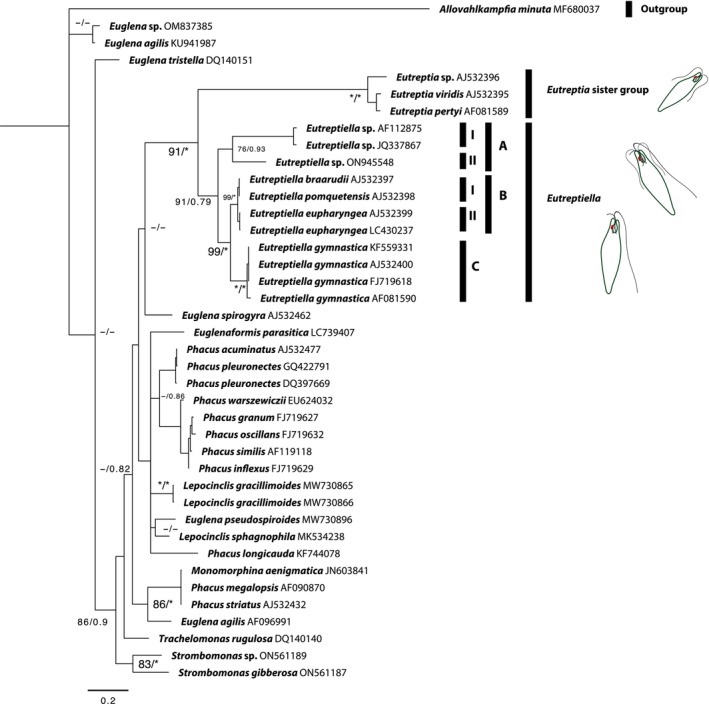
Bayesian inference (BI) topology based on 18S sequence data. Values on branches are BI values (right) and maximum likelihood (ML) bootstrap (left). Asterisks indicate full support (ML = 100%, BI = 1.0%); dashes indicate values below 70%. Scale bar: BI.

Reviewing genetic sequences and additional phylogenetic analyses are necessary to clarify these relationships and better understand the evolution and diversity within the genus *Eutreptiella*. According to Figure 2, which considered all records of *Eutreptiella* and *Eutreptia* species with 18S (SSU) phylogenetic information (Table 1), it is confirmed that *Eutreptia* and *Eutreptiella* are sister genera and form a monophyletic group within the order Eutreptiales consistent with the findings of Marin et al. (2003). Strictly within the species of *Eutreptiella* the group A (sub‐groups I and II) includes several unidentified species (*Eutreptiella* sp. ON945548 (Marin et al., 2003), *Eutreptiella* sp. JQ337867 (Kang et al., 2023) and *Eutreptiella* sp. AF112875 (Kuo & Lin, 2013)), suggesting a diversity of lineages that have yet to be fully characterized. The high genetic variability within this sub‐group indicates the possibility of recent diversification or adaptations to different ecological niches. This diversification suggests that there are many species within these two sub‐groups that still need to be studied and formally described to better understand their evolution and ecology.

**TABLE 1:** Molecular sequences used in this study.

Species	Collection data (country: Site; collector(s); data)	18S GenBank accession	References
*Euglena agilis*	USA: Oregon, Nye Beach	KU941987	–
*Euglena agilis*	New Jersey, USA	DQ140140	Bjørnland (1982)
*Euglena formisparasitica*	Japan: Ibaraki, Tsukuba, Kurihara; Koichiro Kato; 2022‐06‐22	LC739407	Chaber et al. (2022)
*Euglena pseudospiroides*	–	MW730896	Triemer et al. (2006)
*Euglena* sp.	–	OM837385	–
*Euglena spirogyra*	–	AJ532432	Marin et al. (2003)
*Eutreptia pertyi*	–	AF081589	–
*Eutreptia* sp.	–	AJ532396	Marin et al. (2003)
*Eutreptia viridis*	–	AJ532395	Marin et al. (2003)
*Eutreptiella braarudii*	–	AF112875	Linton et al. (1999)
*Eutreptiella eupharyngea*		AJ532398	Marin et al. (2003)
*Eutreptiella eupharyngea*	Japan	LC430237	Yamasaki et al. (2019)
*Eutreptiella gymnastica*		AJ532400	Marin et al. (2003)
*Eutreptiella gymnastica*		KF559331	Szabová et al. (2014)
*Eutreptiella gymnastica*		FJ719618	Kim et al. (2010)
*Eutreptiella gymnastica*	–	AF081590	–
*Eutreptiella pomquetensis*	–	AJ532397	Marin et al. (2003)
*Eutreptiella* sp.	–	AJ532462	Marin et al. (2003)
*Eutreptiella* sp.	–	ON945548	Kang et al. (2023)
*Eutreptiella* sp.	–	JQ337867	Kuo and Lin (2013)
*Lepocinclis gracillimoides*	–	MW730865	Triemer et al. (2006)
*Lepocinclis gracillimoides*	–	MW730866	Triemer et al. (2006)
*Lepocinclis sphagnophila*	–	MK534238	Kim et al. (2013)
*Monomorphina aenigmatica*	–	AF096991	–
*Phacus acuminatus*	–	AJ532477	Marin et al. (2003)
*Phacus granum*	–	FJ719627	Kim et al. (2010)
*Phacus inflexus*	–	FJ719629	Kim et al. (2010)
*Phacus longicauda*	–	KF744078	–
*Phacus megalopsis*	–	JN603841	Yoshikawa (1986)
*Phacus oscillans*	–	FJ719632	Kim et al. (2010)
*Phacus pleuronectes*	–	DQ397669	Łukomska‐Kowalczyk et al. (2020)
*Phacus pleuronectes*	–	GQ422791	Kato et al. (2023)
*Phacus similis*	–	AF119118	–
*Phacus striatus*	–	AF090870	–
*Phacus warszewiczii*	–	EU624032	–
*Strombomonas gibberosa*	China	ON561189	–
*Strombomonas* sp.	New Jersey, USA	DQ140151	Bjørnland (1982)
*Trachelomonas rugulosa*	China	ON561187	–

Group B of the genus *Eutreptiella* in the cladogram, includes in the sub‐group I the species *Eutreptiella braarudii* (Linton et al., 1999) and *E. pomquetensis* (Marin et al., 2003); the sub‐group II shows a repetition of the species *E. eupharyngea* (AJ532399 (Marin et al., 2003), LC430237 (Yamasaki et al., 2019)). The genetic proximity between these species suggests that they may have recently diverged from a common ancestor, which could indicate a recent speciation process where genetic differences are not yet pronounced enough to clearly separate them considering genetic distances with a value of <0.007 (Table 2). It is important considered that species delimitation within the order Eutreptiales using the 18S rRNA gene, typically relies on comparing genetic distances between sequences; in other genera of euglenoids (e.g., *Discoplastis* Triemer) the interspecific variation is 2.9% (Łukomska‐Kowalczyk et al., 2021). Although there is no universally accepted threshold, studies in other groups suggest that a genetic distance of minimum 3.2% generally indicates the presence of distinct species (Escarcega‐Bata et al., 2021). This ≈3% threshold is commonly accepted in 28S rRNA; however, the 18S rRNA gene is more conserved, leading to the acceptance of smaller variations, as seen in diatoms with a minimum of 1% dissimilarity (Moniz & Kaczmarska, 2009). In Euglenoids, this threshold can vary depending on the specific group of organisms and the context of the study.

**TABLE 2:** Table of p‐distance used in this study.

	*Allovahlkampfia minuta* MF680037	*Eutreptiella braarudii* AJ532397	*Eutreptiella pomquetensis* AJ532398	*Eutreptiella eupharyngea* AJ532399	*Eutreptiella eupharyngea* LC430237	*Eutreptiella gymnastica* KF559331	*Eutreptiella gymnastica* AJ532400	*Eutreptiella gymnastica* FJ719618	*Eutreptiella gymnastica* AF081590
*Allovahlkampfia minuta* MF680037									
***Eutreptiella braarudii*** **AJ532397**	0.2948905109								
***Eutreptiella pomquetensis*** **AJ532398**	0.2934306569	**0.0043795620**							
***Eutreptiella eupharyngea*** **AJ532399**	0.2948905109	**0.0072992701**	**0.0029197080**						
***Eutreptiella eupharyngea*** **LC430237**	0.2948905109	**0.0072992701**	**0.0029197080**	**0.0000000000**					
*Eutreptiella gymnastica* KF559331	0.3051094891	0.0496350365	0.0467153285	0.0452554745	0.0452554745				
*Eutreptiella gymnastica* AJ532400	0.3051094891	0.0496350365	0.0467153285	0.0452554745	0.0452554745	0.0000000000			
*Eutreptiella gymnastica* FJ719618	0.3051094891	0.0496350365	0.0467153285	0.0452554745	0.0452554745	0.0000000000	0.0000000000		
***Eutreptiella gymnastica*** **AF081590**	0.3051094891	0.0525547445	0.0496350365	0.0496350365	0.0496350365	0.0072992701	0.0072992701	0.0072992701	

*Note*: Distances within clade B are highlighted in blue, and those within clade C are highlighted in green.

A remarkable issue in Eutreptiales, and euglenoids in general, is the reliance solely on the 18S rRNA gene for species delimitation. For instance, while sequences for the 28S and ITS rRNA gene exist for *Eutreptiella gymnastica* (Hrdá et al., 2012), the lack of comparative sequences makes its use impractical. Chloroplast genes, due to their conserved mutation rates and similarity to chlorophyll‐related genes, provide an alternative but are influenced by their acquired nature. Mitochondrial genes, however, often present complications due to the unique evolutionary origin of Eutreptiales. This multifaceted approach highlights the need for incorporating multiple genetic markers and considering the evolutionary context to achieve accurate species delimitation in Eutreptiales, similar to the issues encountered when comparing various species of algae (Leliaert et al., 2014). This close grouping could also reflect significant intraspecific variability, where the observed differences are variations within the same or sister species and these groups would have to be amended, providing that it is accompanied by morphological information.

However, it is also possible that the species are misidentified in databases like GenBank due to taxonomic identification errors. In some cases, the genetic sequences used to construct the phylogenetic tree may not be suitable for resolving relationships at this level, resulting in the incorrect grouping of unrelated species, causing polyphyly within the group. This could indicate that the observed similarities between *Eutreptiella braarudii* and *E. pomquetensis* are not real but the result of identification errors, as seen in the origin of the sequences (Table 1). All related articles have the molecular basis and/or do not include a morphological analysis study for their identification (e.g., Marin et al., 2003). Additionally, it is probable that these issues are due to insufficient sampling of *Eutreptiella* species.

Undersampling presents a significant challenge in accurately understanding the genetic diversity and evolutionary relationships within *Eutreptiella*. When species are underrepresented in genetic databases, it leads to an incomplete characterization of the genus. In the case of *Eutreptiella*, many sequences in GenBank are identified only at the genus level, without precise species identification (Table 1). This gap in data makes it impossible to establish reliable phylogenetic relationships and, consequently, to interpret evolutionary processes. Moreover, the scarce sampling and incomplete species identification issues observed in *Eutreptiella* highlight the needed of including morphological data for accurate identification. Comprehensive sampling efforts, including both morphological and molecular analyses, are essential to address the gaps in our understanding and to accurately determine the phylogenetic relationships within this group. Additionally, it opens the possibility that the genus is polyphyletic, as often happens within euglenoids (Marin et al., 2003), requiring more in‐depth studies combining holistic approaches.

The issue of scarce sampling is compounded by the fact that marine euglenoids, in general, are not as extensively studied as other groups. For instance, the genus *Eutreptia*, a sister group to *Eutreptiella*, also suffers from limited genetic studies. Despite there being 11 accepted species of *Eutreptia* (Guiry & Guiry, 2024), only *Eutreptia viridis* Perty 1852 (AJ532395) and *Eutreptia pertyi* Pringsheim 1953 (AF081589) have undergone any molecular work (18S) (Marin et al., 2003). This lack of comprehensive sampling and molecular analysis makes it challenging to draw accurate conclusions about their evolutionary history and relationships.

Group C with multiple accessions of *Eutreptiella gymnastica* (AJ532400 (Marin et al., 2003), KF559331 (Szabová et al., 2014), FJ719618 (Kim et al., 2010), AF081590). The AF081590 accession shows a p‐distance of 0.0072992701 relative to the others. However, it cannot be conclusively identified as *E. gymnastica* since it was not published in a peer‐reviewed article, leaving its identity unverified due to the absence of descriptions or images.

## 4 GENOMICS AND MOLECULAR BIOLOGY

The study of the plastid genome of *Eutreptiella* species has provided valuable information about the process of secondary endosymbiosis in euglenoids. The complete chloroplast genome sequencing of *E. gymnastica* revealed a length of 67,622 base pairs, which allows tracing gene transfer and loss events. This comparative analysis has shown a significant reduction in the gene repertoire from its common ancestor with other photosynthetic euglenoids (Dabbagh et al., 2017; Hrdá et al., 2012).

Additionally, transcriptomic studies have identified complex carbon fixation mechanisms and a conserved spliced leader sequence (Eut‐SL) in the mRNAs of *Eutreptiella* sp., suggesting an advanced regulatory system and specific adaptations for cell division control and lipid production (Kuo et al., 2021). These studies have revealed the presence of mRNA sequences with a high prevalence of leader splicing, indicating a complex gene regulation and adaptive expression mechanism.

Genome and transcriptome analysis of *Eutreptiella* species has allowed the identification of genes involved in photosynthesis, lipid metabolism, and environmental stress response. These studies have shown that these species possess a significant capacity to adapt their metabolism to different environmental conditions, which is crucial for their survival in variable marine environments (Dickinson, 2008).

## 5 DISTRIBUTION AND HABITAT

*Eutreptiella* is a genus of euglenoid algae that primarily inhabits marine and brackish environments. These microalgae are cosmopolitan, found in various regions around the world, including coastal zones and estuaries. Their presence is common in waters with high nutrient availability, which favors their growth and proliferation. *Eutreptiella* can form massive blooms under nutrient enrichment conditions, a phenomenon observed in various geographical locations (Acedo et al., 2005; Anderson et al., 2002; Du Yoo et al., 2018; Yamasaki et al., 2019).

From 1999 to 2005, *Eutreptiella* sp. formed a massive bloom along the San Marcos beach, Canary Islands (Acedo et al., 2005). The analysis of the geographical distributions of *Eutreptiella* has revealed that these algae prefer nutrient‐rich waters and are particularly abundant in coastal upwelling areas and estuaries. These zones provide ideal conditions for rapid growth and species proliferation, highlighting their importance in nutrient cycling and primary production in these ecosystems (Du Yoo et al., 2018; Olli et al., 1996).

**FIGURE 3:**
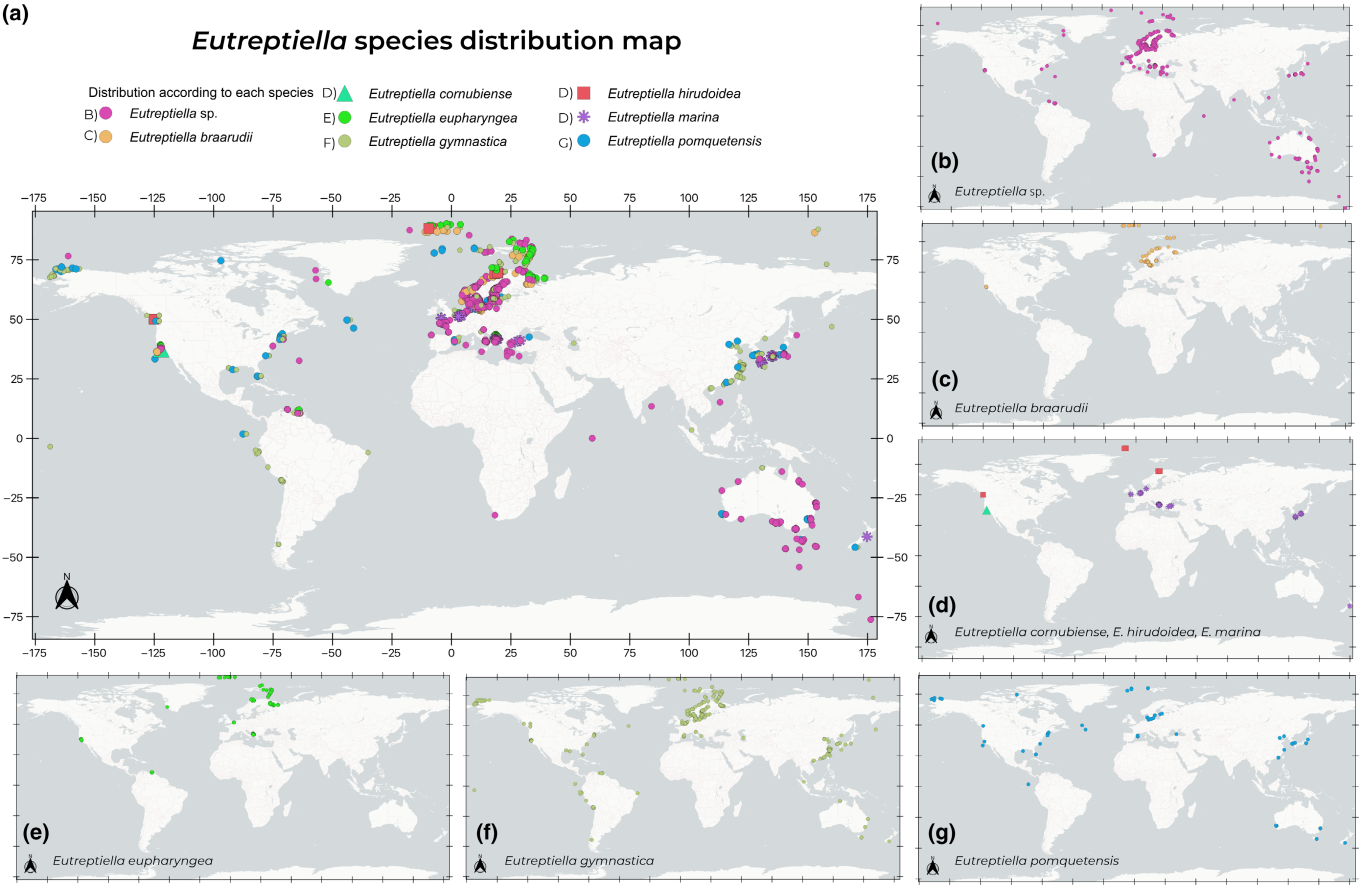
(a) Distribution of various species of the genus *Eutreptiella* worldwide. Each species is represented by points indicating the georeferenced locations where they have been found. (b) Map of *Eutreptiella* sp. distribution. (c) Map of *E. braarudii* distribution. (d) Map of *E.cornubiense* (triangle) *E. hirudoidea* (square) and *E. marina* (asterisk) distribution. (e) Map of *E. eupharyngea* distribution. (f) Map of *E. gymnastica* distribution. (g) Map of *E. pomquetensis* distribution.

Figure 3 shows the worldwide distribution of several species of *Eutreptiella* using information from various scientific databases (Table 3). Each species is represented by different colored or symbology points indicating the locations where they have been found. The biogeographical behavior is described below:

*Eutreptiella* sp. (Figure 3B): Found in the North Atlantic and some parts of the Pacific Ocean, including the west coast of North America, Japan, and records in all Australia, along with some occurrences in other parts of the world. This category can refer to any species of the genus *Eutreptiella* (7540 records).
*Eutreptiella braarudii* (Figure 3C): Distribution similar to *Eutreptiella* sp., found in the Northern Hemisphere, including, principally Europe, and some areas of North America and one in Asia (497 records).
*Eutreptiella cornubiense* (Figure 3D triangle): It has a specific distribution in the area along the Weast Coast of North America (1 record).
*Eutreptiella hirudoidea* (Figure 3D square): It has very specific locations on the West Coast of Canada, Northern Europe, and in the Arctic (8 records).
*Eutreptiella marina* (Figure 3D asterisk): Distribution in Europe and the Japanese and Korean Pacific (216 records).
*Eutreptiella eupharyngea* (Figure 3E): Founded in the Northern Hemisphere, especially in North America and Europe, with some points in the Caribe (303 records).
*Eutreptiella gymnastica* (Figure 3F): It is distributed throughout the Pacific, with records also in the eastern Atlantic and across Europe (1570 records).
*Eutreptiella pomquetensis* (Figure 3G): Primarily distributed in northern latitudes, with some locations in South America (in the equatorial Pacific Ocean) and in Australia and New Zealand (190 records).

**TABLE 3:** Records of *Eutreptiella* in different databases and GBIF (Material without Data).

Direct observation	
Organization	Number of records
The Norwegian Biodiversity Information Centre (NBIC)	1112
PANGAEA – Data Publisher for Earth & Environmental Science	398
CSIRO National Collections and Marine Infrastructure (NCMI) Information and Data Centre (IDC)	339
Danish Centre for Environment and Energy, Aarhus University	249
United States Geological Survey	209
Dutch Foundation for Applied Water Research	141
IFREMER – French Research Institute for Exploitation of the Sea	132
National Institute of Oceanography and Applied Geophysics	77
Caribbean OBIS Node	115
Observation with material	
The Swedish Meteorological and Hydrological Institute	4925
MGnify	840
UiO Department of Biosciences	126
United States Geological Survey	40
Flanders Research Institute for Agriculture, Fisheries and Food (ILVO)	7
NatureMetrics	1
Curated material	
CSIRO National Collections and Marine Infrastructure (NCMI) Information and Data Centre (IDC)	819
National Museum of Nature and Science, Japan	254
Conservation of Arctic Flora and Fauna	174
Alaska Ocean Observing System	150
Material without data	
Flanders Marine Institute	144
The Nansen Legacy Project	20
Marine Biology Laboratory	8
UiT The Arctic University of Norway	5

Based on this information, most *Eutreptiella* species are primarily distributed in the North Atlantic, with a notable concentration on the coasts of Western Europe and North America, as well as in the Arctic. This concentration suggests greater sampling efforts in these regions, indicating more attention and resources dedicated to research in these areas. Some species are also found in parts of the Pacific Ocean and around Australia and New Zealand, although these points are less numerous.

The lack of points in other parts of the world, such as the Indian Ocean and parts of the South Pacific, South Atlantic and Africa, could indicate a lack of studies and sampling efforts in these regions. This suggests that the global distribution of *Eutreptiella* species might be underrepresented due to the absence of extensive research in these areas.

Regarding their global records (Table 3), the data collection on *Eutreptiella* species reflects a significant effort by various institutions to document and understand their patterns. The category of direct observations accumulates a total of 2772 records, with the Norwegian Biodiversity Information Centre (NBIC, 2024), leading with 1112 records. This highlights the importance of in situ observations for obtaining an initial understanding of species distribution and behavior. Organizations like PANGAEA (2024) and CSIRO (2024) also contribute notably, emphasizing the crucial role of data centers in aggregating environmental information.

As for observations with sampled material, totaling 5939 records, The Swedish Meteorological and Hydrological Institute (2024) is the main contributor with 4925 records. This category is essential as it combines direct observation with physical samples, providing a more robust basis for scientific analysis. The considerable contribution from MGnify (2024) and the UiO Department of Biosciences (2024) also underscores the relevance of data backed by material in scientific research.

Curated material, comprising 1397 records, comes from recognized institutions like CSIRO (2024) and The National Museum of Nature and Science in Japan (2024). These records have been carefully selected and maintained, ensuring their quality and accuracy. This type of data is fundamental for longitudinal and comparative research, providing a stable and reliable data set. The preservation of curated data allows researchers to access historical and contemporary information, facilitating trend, and change studies over time.

Nevertheless, the category of material without data, which includes 177 records, indicates areas where data collection can improve. The lack of associated data with these materials could be due to various factors, such as lack of resources or difficulties in data collection. However, these records can still be valuable in identifying knowledge gaps and guiding future research efforts to fill these voids.

It is important to mention that GBIF (2024) provides detailed information on these data for this study, but records that do not have adequate references or do not come from recognized institutions and/or are not correctly cited are omitted. This approach ensures that the quality and reliability of the data are maintained, thus promoting scientific integrity.

In total, 10,285 records demonstrate a global and coordinated effort in studying *Eutreptiella* species; however, the lack of Latin organizations and/or solid records in articles shows that much research is still needed.

## 6 ECOLOGICAL IMPORTANCE

The blooms of *Eutreptiella* species can have significant effects on local ecosystems. These blooms often occur in spring and summer when light and temperature conditions are optimal for rapid growth. During these blooms, *Eutreptiella* species can dominate the phytoplankton, affecting nutrient availability and altering the dynamics of microbial and phytoplankton communities (Iwasaki, 1971; Jeong et al., 2011; Okumura et al., 2001).

The growth and distribution of *Eutreptiella* species are strongly influenced by environmental factors such as light availability, temperature, salinity, and nutrients. These algae exhibit high phenotypic plasticity, allowing them to adapt to fluctuations in environmental conditions. For example, *Eutreptiella* has been observed to adjust its photosynthesis rate and lipid production in response to changes in light intensity and nutrient availability, thus optimizing its metabolic efficiency (Jeong et al., 2011; Olli et al., 1996).

In terms of salinity, *Eutreptiella* species show broad tolerance, capable of surviving, and growing in conditions ranging from brackish to high‐salinity marine environments. This adaptability is crucial for their ability to colonize different aquatic habitats and form blooms under favorable conditions. Additionally, these species can form dormant cysts in response to unfavorable conditions, allowing them to withstand periods of environmental stress and subsequently germinate when conditions improve (Olli et al., 1996). As primary producers, they contribute significantly to phytoplankton biomass, serving as a food source for a variety of marine organisms, including zooplankton and small fish. Their ability to form blooms can significantly impact the structure and dynamics of planktonic communities, affecting nutrient availability and species composition in the ecosystem (Jeong et al., 2011; Papry et al., 2020).

## 7 ECOLOGICAL AND FUNCTIONAL INTERACTIONS

The interactions of *Eutreptiella* species with other microorganisms in the marine environment are of great importance. Ectobiotic and endobiotic bacteria associated with them can influence their growth and the dynamics of algal populations. These bacteria can provide essential nutrients such as vitamin B12, crucial for the algae's growth and reproduction (Du Yoo et al., 2018; Kuo et al., 2021; Kuo & Lin, 2013).

Ectobiotic bacteria present on the surface of algal cells can form mutualistic associations with *Eutreptiella* species, providing nutrients and bioactive compounds that enhance their growth. However, endobiotic bacteria living inside algal cells can play roles in regulating metabolism and defense against pathogens (Kuo & Lin, 2013). These interactions are essential for understanding their ecology and physiology in a natural habitat. Recent studies have shown that associated bacteria can influence lipid production and the photosynthetic capacity of *Eutreptiella* species, which has implications for their use in biotechnology and biofuel production (Hrdá et al., 2012; Kang et al., 2023; Kuo & Lin, 2013).

## 8 METABOLISM AND FUNCTIONAL CAPACITIES

*Eutreptiella* species have shown remarkable metabolic capacity, including the synthesis of omega‐3 fatty acids, such as alpha‐linolenic acid and docosahexaenoic acid (DHA). These compounds are of great interest due to their health benefits and potential in nutritional supplement production. The high concentration of these fatty acids in *Eutreptiella* highlights their potential for biotechnological applications (Kang et al., 2023; Kuo & Lin, 2013).

The ability of *Eutreptiella* to produce valuable lipids has been the subject of studies seeking to optimize cultivation conditions to maximize the production of these compounds. These studies have demonstrated that factors such as nutrient availability, light intensity, and temperature can significantly influence lipid production, providing a basis for developing efficient cultivation techniques (Kang et al., 2023; Yamasaki et al., 2019). Moreover, these species can accumulate lipids under stress conditions, which can be exploited to increase biofuel production. This stress response capability also underscores their adaptability to different environmental conditions, crucial for their survival and proliferation in marine environments (Kang et al., 2023; Yamasaki et al., 2019).

## 9 BIOTECHNOLOGICAL POTENTIAL

Interest in *Eutreptiella* extends to their potential in biotechnological applications and their ability to produce bioactive compounds with pharmacological properties. These microalgae are promising sources of bioactive compounds, biofuels, and nutritional supplements due to their ability to produce omega‐3 fatty acids and other valuable lipids. Studies have explored their use in biofuel production and improving aquaculture diets, demonstrating their versatility and utility in various industries (Iwasaki, 1971; Kang et al., 2023; Kuo & Lin, 2013). Recent research has shown that extracts from *Eutreptiella* species possess antioxidant and anti‐inflammatory properties, suggesting their potential for developing new drugs and health supplements (Kang et al., 2023). These bioactive compounds can be used in the pharmaceutical industry to develop treatments for a variety of inflammatory and degenerative diseases.

*Eutreptiella* species are promising candidates for biofuel production due to their high lipid productivity. Cultivation conditions can be optimized to increase lipid accumulation, which can be used for biodiesel production. This ability to accumulate lipids under stress conditions is a valuable characteristic that can be exploited in industrial processes (Okumura et al., 2001; Yamasaki et al., 2019). Research in this area continues with the aim of improving the efficiency and sustainability of biofuel production from microalgae.

The biotechnology of microalgae, including *Eutreptiella* species, offers a sustainable and environmentally friendly approach to producing valuable products. Microalgae can be cultivated in various environments, including closed and open systems using seawater and wastewater, minimizing competition for land and freshwater resources. This highlights the potential of *Eutreptiella* not only as a source of valuable products but also as an integral part of biotechnological solutions to environmental and energy challenges (Kang et al., 2023; Okumura et al., 2001).

## 10 CONCLUSION

*Eutreptiella* is a diverse and ecologically significant genus of euglenoid algae that play a crucial role in marine ecosystems. Their ability to adapt to different environments, interact with bacteria, and their biotechnological potential make them an important subject of study in marine and biotechnological research. As research progresses, it is expected to discover more details about their molecular biology, genomics, and practical applications. The continuous exploration of their metabolic capacities and ecological interactions will provide new opportunities for industrial and environmental applications. Understanding and managing the populations of *Eutreptiella* species is essential to harness their potential and ensure the health of marine ecosystems, highlighting their importance in the future of biotechnology.

The case of *Eutreptiella* is very interesting, where at the molecular level, the issues are not resolved, leading to several hypotheses about why there is such species variability within such a small genetic distance. Therefore, the next step is to conduct comparisons and holistic studies.

It is also important to note that there may be misidentifications within *Eutreptiella* species, or it may be a polyphyletic group. This suggests the need for further comprehensive studies to clarify the taxonomy and evolutionary relationships within the genus.

This study, rather than resolving issues, raised more questions about the group and euglenoids. Another perspective would be to study the sister group *Eutreptia* and consider its implications within the broader issues affecting all these organisms.

## AUTHOR CONTRIBUTIONS

**Sinuhé Hernández‐Márquez:** Conceptualization (equal); data curation (equal); formal analysis (equal); investigation (equal); methodology (equal); software (equal); validation (equal); writing – original draft (equal). **María Eugenia Zamudio‐Resendiz:** Data curation (equal); project administration (equal); resources (equal); software (equal); supervision (equal); validation (equal); writing – review and editing (equal). **Montserrat Murrieta‐Alarcón:** Software (equal); visualization (equal); writing – review and editing (equal). **María Luisa Nuñez‐Resendiz:** Conceptualization (equal); formal analysis (equal); methodology (equal); resources (equal); software (equal); supervision (equal); validation (equal); writing – review and editing (equal). **Kurt Martin Dreckmann:** Funding acquisition (equal); resources (equal); validation (equal); writing – review and editing (equal). **Abel Sentíes:** Resources (equal); supervision (equal); validation (equal); writing – review and editing (equal).

## FUNDING INFORMATION

This work was supported by the projects: UAMI‐CBS2023‐2026: session 84.22‐211222 and UAMI‐CA‐117.

## CONFLICT OF INTEREST STATEMENT

The authors declare that they have no known competing financial interests or personal relationships that could have appeared to influence the work reported in this paper.

## CODE AVAILABILITY

Qgis 3.36.3 in https://www.qgis.org/. Geneious prime in https://www.geneious.com/. jModelTest in https://github.com/ddarriba/jmodeltest2. GenBank in https://www.ncbi.nlm.nih.gov/genbank/.

## Supporting information


Data S1–S2.

## Data Availability

The data are available in Table 1 and in the Appendix 1.

## APPENDIX 1

### Phylogenetic analysis method

The alignment, including sequences of all species of *Eutreptiella* available in GenBank for 18S (Table 1), was performed using Geneious prime. *Allovahlkampfia minuta* was used as the outgroup. Phylogenetic analyses were conducted using Bayesian inference (BI) and maximum likelihood (ML). The selected evolutionary model was GTR + G + I (general time reversible + gamma distribution + invariable sites) determined on the jModelTest. ML analysis was performed using Geneious prime. Support for each branch was obtained from 1000 bootstrap replications. BI analysis was performed using MrBayes v3.2.2 (Ronquist et al., 2012). Four chains of Markov chain Monte Carlo were used, starting with a random tree and sampling the data every 500 generations for 5 × 106 generations. The posterior probability support for clades were established after 1,000,000, but we discarded 25% of the trees as burn‐in. Pairwise distance values (p distances) were calculated using Geneious prime.
